# Human Campylobacteriosis Cases Traceable to Chicken Meat—Evidence for Disseminated Outbreaks in Finland

**DOI:** 10.3390/pathogens9110868

**Published:** 2020-10-22

**Authors:** Ann-Katrin Llarena, Rauni Kivistö

**Affiliations:** 1 Food Safety Unit, Department of Paraclinical Sciences, Faculty of Veterinary Medicine, Norwegian University of Life Sciences, 1430 Ås, Norway; ann-katrin.llarena@nmbu.no; 2 Department of Food Hygiene and Environmental Health, Faculty of Veterinary Medicine, University of Helsinki, FI-00790 Helsinki, Finland

**Keywords:** *Campylobacter jejuni*, whole-genome sequencing, epidemiology, food safety, chicken meat

## Abstract

*Campylobacter jejuni* (*C. jejuni*) is the most common cause of human bacterial gastroenteritis in the world. Food-borne campylobacteriosis is thought to be commonly caused by the handling and consumption of undercooked chicken meat, but the epidemiology of this disease is complex and remains poorly characterized, especially in the Nordic countries. Here, we used state-of-the-art methods in genetic epidemiology combined with patient background and temporal association data to trace domestically acquired human *C. jejuni* infections (*n* = 50) to chicken meat, in a midsize Nordic town in Finland during a seasonal peak. Although 59.2% of the human isolates shared a sequence type (ST) with a chicken batch slaughtered prior to the onset of disease, further analysis at the whole-genome level (core genome and whole-genome multilocus sequence typing, cgMLST and wgMLST, respectively) traced a mere nine cases (18.4%) to fresh chicken meat. Human isolates also shared genotypes with isolates collected from chicken batches slaughtered after the onset of the human disease, highlighting the role of alternative transmission pathways from chickens to humans besides the food chain, or a shared third source. The high resolution offered by wgMLST, combined with simple metadata, offers a more accurate way to trace sporadic cases to possible sources and reveal disseminated outbreak clustering in time, confirming the importance of complementing epidemiological investigations with molecular epidemiological data.

## 1. Introduction

Chicken meat is a sustainable food option with a small carbon footprint of its production relative to other livestock [1]. The production and consumption of chicken meat has been increasing in Finland and abroad [2]. Broiler chicken is a reservoir for the human pathogen *Campylobacter*, the most common cause of human bacterial gastroenteritis worldwide with an estimated cost of 2.4 billion euros annually in the European Union (EU) [3]. Reducing the number of campylobacteriosis cases is therefore of high priority for stakeholders. According to the European Food Safety Authority (EFSA) and World Health Organization (WHO), the number of campylobacteriosis cases can be efficiently reduced by lowering the number of *Campylobacter*-positive chicken flocks [3,4,5]. The food industry and public health authorities spend large resources on surveying and reducing *Campylobacter* spp. colonization in poultry, such as through The Finnish Monitoring Program (FMC) for *Campylobacter* in broilers [6,7]. According to FMC, every chicken batch slaughtered between June and October is tested for the presence of *C. jejuni* and *C. coli* at slaughter, although no action for the chicken meat after a positive result is taken.

Similarly to the remainder of the Nordic hemisphere, Finland has a clear peak in the number of human campylobacteriosis cases during the summer [8], and the role of broiler chickens in the epidemiology of campylobacteriosis is not completely understood in the Nordic countries [3,9]. European surveys suggest that the chicken reservoir is the origin of more campylobacteriosis cases than the consumption of chicken meat alone can explain [3], implying that there are other transmission routes for *Campylobacter* from chicken flocks to humans. Traditional epidemiology, in the form of case-control studies [10,11], provides valuable insight to the associated risk factors of campylobacteriosis, although combining genomic and temporal data of *C. jejuni* isolates from chickens and humans has the potential to trace the bacteria back to a possible origin through comparative genomics. When performing such genomic tracing or epidemiological studies, robust sampling is necessary to capture the whole diversity of isolates circulating in the populations being compared. One such study by Kovanen et al. (2016), showed through the use of whole-genome sequencing (WGS) that only one in five campylobacteriosis cases in three Finnish hospital districts was traceable to the Finnish chicken reservoir, and revealed a clustering of human cases without a possible coupling to chicken isolates [12]. Such diffuse outbreaks, i.e., the occurrence of temporal and/or spatial clusters of genotypically similar isolates among apparently sporadic cases, are suggested to be several times more common than point-source outbreaks for *Campylobacter* infections [13,14,15]. More research is needed to show if such a distribution of cases is a trend or a one-time event.

Increased efforts to elucidate the relative contribution of different sources and pathways for human infection are valuable, and ongoing surveillance is essential if the impact of intervention programs on human disease burden are to be assessed accurately. Stakeholders are dependent on this type of knowledge to design and evaluate cost-efficient mitigation strategies, such as freezing or heat-treatment, to reduce the number of campylobacteriosis cases. Here, we use WGS to detect diffuse outbreaks among presumptive sporadic campylobacteriosis cases during a summer peak. Furthermore, we aim to trace both sporadic and diffuse outbreaks of human cases to the chicken reservoir to answer two main research questions; (1) How many campylobacteriosis cases during the seasonal peak are possibly due to the consumption of Finnish chicken meat? (2) How common are diffuse outbreaks during the Finnish campylobacteriosis summer peak? To do so, we subjected human and chicken *C. jejuni* isolates to WGS and analysis using pipelines developed by the INNUENDO project [16] and PopPUNK [17].

## 2. Results

### 2.1. Dataset

**Figure 1:**
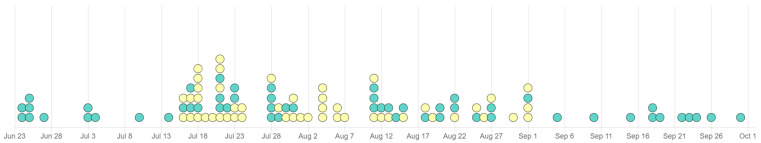
Timeline of sample collection of *Campylobacter jejuni*-positive samples (absolute numbers, *y*-axis) from chicken (turquoise) and human cases (yellow) during a summer peak in Finland. Timeline produced in Microreact [19].

To answer if chicken meat is a possible source of human campylobacteriosis cases, *C. jejuni* isolates from chickens and humans acquired during a seasonal peak were selected and compared. The criteria for the inclusion of chicken isolates (temporal association: chicken batch slaughtered 2–23 days prior to human case) resulted in the selection of 39 *C. jejuni* isolates collected from chicken flocks slaughtered between 24 June 2014 and 30 September 2014 (Figure 1). Four of the chicken isolates acquired on the same day or after the sampling of the last human case were also included to account for transmission pathways other than food. Together with the 50 human isolates, the dataset consisted of 89 *C. jejuni* isolates subjected to WGS. Of these, one human isolate was identified as *C. upsaliensis* using the 7-loci multilocus sequence typing (MLST) schema. Therefore, 104 human and chicken *C. jejuni* isolates were included in the downstream analysis (Table S1). The majority of the assemblies are available from Zenodo [18], and raw reads have been submitted to the European Nucleotide Archive (ENA) under project PRJEB27020, while the genome assemblies of five chicken strains are available from PubMLST (id: 106378-106382).

### 2.2. Identifying Clusters of Human and Chicken Isolates

Genotyping was performed using *k-mer*-based and gene-by-gene analysis to compare the human and chicken isolates: the 7-loci multilocus sequence typing (MLST) [20], the INNUENDO core genome MLST (cgMLST) schema [16,18], whole-genome MLST (wgMLST) [21] and PopPUNK population structure [17]. Isolates with a shared genotype are hereafter referred to as clusters, and if they shared a temporal association (see above), they were considered to be epidemiologically linked. On the 7-loci MLST level, several clusters of chicken and human isolates were evident (Table 1), of which the majority were of the sequence type (ST) 45; 18 of 19 human ST-45 isolates were temporally linked to one or more chicken ST-45 isolates. Five ST-677 and three ST-267 human isolates were genotypically and temporally linked to an ST-677 and ST-267 chicken isolate, respectively, as was one human isolate each of ST-19, ST-21 and ST-230. In total, we traced 59.2% (*n* = 29/49) of the human cases to the chicken reservoir using 7-loci MLST genotyping.

**Figure 2:**
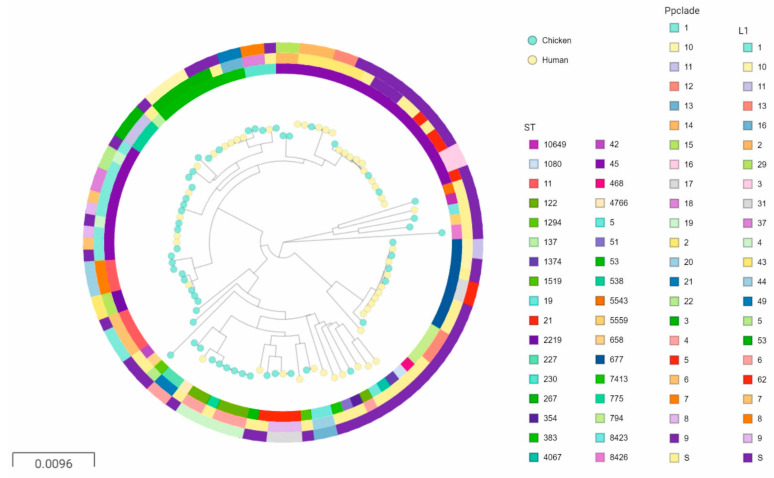
A neighbor joining tree of the *k-mer* distances of the core genome of 49 human isolates (yellow nodes) and 55 chicken isolates (turquoise nodes) rooted at the midpoint. STs are indicated in the inner ring (see legend), PopPUNK clade (PPclade) in the middle ring and core loci (L1-cgMLST678) profile in the outer. S denotes PPclades and L1_cgMLST678 profiles represented with only a single isolate. An interactive tree with all metadata is available at https://microreact.org/project/oP3ZG4Szq9EVwsBNJF8eyk/58ac2108 [19].

To assess if these clusters indeed were populated by the same clone, the isolates were compared using two principally different methods: the rapid distance-based *k-mer* population structure analysis using PopPUNK, and two gene-by-gene approaches; the INNUENDO cgMLST and wgMLST schema [18] using the chewBBACA suite [21]. A fitted PopPUNK model with a high score (>0.9) and low density (~0.02) was considered specific, although the recombining nature of *C. jejuni* somewhat erased the population structure and resulted in the formation of many clades (*n* = 54), of which 33 contained only a single isolate (Figure 2). There was a high concordance between ST and PopPUNK clades (PPclades), and PopPUNK had a higher resolution because it split STs into several PPclades. Within PPclade 1, 2, and 3, three, five and three human isolates could be traced back to chicken isolates slaughtered 2–23 days prior to the sampling of the human case(s), respectively. In addition, one human isolate each belonging to seven PPclades (6, 8, 10, 17, 18, 19, and 20) could be traced to a chicken flock of the same PPclade with a temporal association. Taken together, based on PPclades, 18 of the 49 human isolates (36.7%) could have passed from the chicken reservoir to humans through the handling or eating of fresh chicken meat. 

To investigate if the PPclades contained truly clonal isolates, we adopted the cgMLST678 schema with validated and robust cut-off values for clonality as defined by the INNUENDO consortium [16]. Here, isolates were considered clones if they were similar in at least 674 of 678 core loci (hereafter referred to as L1-cgMLST678 types). According to this analysis, the *C. jejuni* population circulating among chickens and humans consisted of 72 different L1-cgMLST678 types. The application of L1-cgMLST678 for genotyping and cluster definition reduced the number of human and chicken clusters from the 18 identified by PopPUNK, to a total of ten human cases (ST-19 (*n* = 1), ST-21 (*n* = 1), ST-45 (*n* = 2), ST-230 (*n* = 1), ST-267 (*n* = 2), ST-523 (*n* = 1) and ST-677 (*n* = 2)) (Table 2).

To increase the resolution and test the robustness of these clusters, cgMLST99 (994 loci analyzed) and cgMLST95 (1013 loci analyzed) deducted by the chewBBACA suite from the wgMLST INNUENDO, wgMLST schema were applied to all isolates. Clonal isolates were allowed to vary at a maximum of six loci, which corresponds to 0.59% loci variation within an outbreak, as depicted by the INNUENDO consortium. The increased resolution broke four of the clusters identified by the L1-cgMLST678 analysis (footnote c in Table 2). In fact, the nine human isolates with similar 95cgMLST and 99cgMLST profiles with chicken isolates were extremely similar even on the wgMLST level: three human isolates were identical to their chicken batch equivalents, while four or fewer loci variations were observed for six human isolates and their chicken isolate partners over a total of 2809 loci in the wgMLST analysis. To conclude, we traced 9 of 49 human isolates to one or more chicken flock(s) (18.4%) using a combination of cgMLST and wgMLST analysis.

Four clusters of chicken and human isolates for which the human isolate was collected before or simultaneously with the chicken isolate(s) were also discovered (Table 2); in three of these, the human isolates were collected within three weeks prior to the chicken isolate. In addition, one cluster where the human and chicken isolate was collected more than a month apart was also discovered. Seven clusters containing only chicken isolates collected within two weeks of each other were observed, and two clusters containing only human isolates were also collected during the same week. Nine of these clusters were also grouped using cgMLST99 and cgMLST95 profile analysis (footnote b in Table 2).

## 3. Discussion

Here, we traced nine human campylobacteriosis cases (18.4%) occurring during a summer peak to fresh chicken meat (28.5% potentially with the chicken reservoir) using WGS analysis and one single metadata value, i.e., the date of collection for the human and chicken sample. If chicken meat was a source for human campylobacteriosis, contaminated meat must have been available for the consumer prior to the onset of illness and the human and chicken isolates must be of a similar genotype. The wide time range used here to define temporal association between the human and chicken *C. jejuni* isolates allowed time from slaughter, time-on-market, incubation time and some delays in sample collection, and was designed to minimize the risk of missing potential links between chicken meat and human cases. Taken together, the simultaneous use of temporal and genotypical associations made estimations of proportions of human cases caused by consumption of chicken meat achievable.

In genomic epidemiology, a shared genotype between two hosts is typically a result of: (1) one host being the source of the *C. jejuni* directly or indirectly for the other; (2) the *C. jejuni* being acquired from a common third source; or (3) the genetic variation for the genotype being so limited that isolates appeared similar even in the absence of an epidemiological link. Using temporal association, we assumed that the chicken reservoir was more likely the source of human disease if the chicken isolate was collected prior to the human isolate. With this principle, we traced 18.4% of our human cases to chicken meat. An earlier study traced ~24% of the campylobacteriosis cases in three Finnish cities to one or more chicken batches, which correlates nicely with our findings (24% vs. 18%, Chi-square, *p* = 0.38) [12]. Furthermore, our findings are in line with the EFSA’s Panel on Biological Hazards (BIOHAZ) opinion on chicken meat being the origin of 20% to 30% of human cases in the EU [3]. We did, however, observe four clusters of human and chicken isolates where the chicken isolate was collected after or simultaneously with the human isolate. One explanation could be that the chicken isolate was transferred to humans through pathways other than through the food chain, which is in line with the findings of the BIOHAZ scientific opinion on chicken: the majority of European campylobacteriosis cases (50–80%) originate from the chicken reservoir but reach humans through pathways other than chicken meat. Exactly how the chicken reservoir contributes to the epidemiology is unknown, but contamination from chicken flocks can readily reach humans indirectly through the environment, other animals and food, as chicken flocks contaminate their surroundings *én masse* when they are *Campylobacter*-positive (as reviewed in [3]). Indeed, risk factors for contracting campylobacteriosis, in addition to the consumption of poultry meat, include drinking non-disinfected water, swimming outdoors, and contact with pets and bovines [11,23,24,25,26]. It is therefore still possible that the origin of the cases (*n* = 5) in these four clusters were the chicken reservoir, but that the transmission occurred through pathways other than the food chain.

We also found eight clusters consisting of only chicken (six) or human isolates (two) (Table 2). The isolates of the solely human clusters were collected within the same week in the same city and were most likely examples of small undiscovered outbreaks with origins other than Finnish chicken meat. More data, such as household data, could elucidate their epidemiology. The isolates in the solely chicken clusters could have originated from chicken batches that shared a common source, or alternatively one chicken flock contaminated the other. However, these chicken isolates were collected from batches originating from different farms, and although transmission between farms is not impossible, it is unlikely because thinning, a common transmission risk between farms [27], is not allowed in Finland. Therefore, a shared common source is plausible, especially considering the simultaneous temporal association that some clusters showed. We cannot exclude, however, that the isolates making up these observed clusters belonged to lineages of *C. jejuni* with limited genetic diversity. Low genetic diversity within lineages makes it very challenging to distinguish between epidemiologically and non-epidemiologically linked isolates, as even high-resolution genotyping is unable to separate between such isolates. Indeed, six of twenty observed clusters in this study were of the generalist ST-45, a lineage known to contain subpopulations of monomorphic clones [28]. The use of descriptive and epidemiological data would improve our ability to differentiate epidemiologically linked isolates from monomorphic clones.

Tracing human isolates to the chicken reservoir is based on the assumptions that the entire genetic diversity of *C.jejuni* circulating among Finnish broilers during the summer is captured, and that the *C. jejuni* in the ceca of broiler chickens is the same as the isolate present on the carcass of one of the flock. There is substantial evidence for both. The vast majority of fresh chicken meat available to consumers is domestically produced [29], and because the FMC tests all chicken batches slaughtered in Finland from May to October, the isolates used here represents the vast majority of the *C. jejuni* circulating among broilers in Finland during the summer of 2014. Furthermore, as the *C. jejuni* from the ceca contaminates the meat during the mechanical slaughter process, the genotype of the *C. jejuni* in the ceca is similar to the genotype present on the meat [30]. However, although ten ceca are taken from each flock at random intervals to ensure representative testing of each flock, only one colony is taken for further analysis. This could lead to an inferior representation of the genetic diversity present in the chicken *C. jejuni* population, and therefore an underestimation of the overlap between chicken and human isolates. Earlier studies have found that co-colonization of chicken flocks is unlikely in Finland, as isolates collected from the same flock were similar when typed by pulsed-field gel electrophoresis (PFGE) (Hakkinen and Kaukonen, 2009, presented at the 15th International Workshop on *Campylobacter*, *Helicobacter* and Related Organisms, Niigata, Japan, September 2–9). Even *C. jejuni* collected from separate chicken houses on the same farm were of the same PFGE-type, MLST and wgMLST type in 76.3% of the cases over a five-year period in Finland [12,25]. *C. jejuni* is horizontally introduced to chicken houses, and the biosecurity level in Finnish poultry farms is high. Coupled with a lower environmental load of *C. jejuni* due to colder winters giving rise to “winter breaks” in the *Campylobacter* circulation [31], prevalence of *Campylobacter* in Finnish chicken flocks is low. Therefore, it is unlikely that two different introduction events would happen during a rearing cycle and the chicken isolates presented here most probably represents the entire genetic diversity available from chicken meat during the summer of 2014. Contrary to this, it is highly unlikely that the entire genetic variation of *C. jejuni* from domestically acquired campylobacteriosis cases is captured. Underreporting of campylobacteriosis cases is common [32], as patients with uncomplicated gastroenteritis seldom seek medical care, and if they do, the doctor might be reluctant to collect a stool sample. As a result, our human strain collection is probably just the tip of the iceberg.

Several tools have been developed to compare the WGS of bacteria and assign clonality. Here, we used the rapid *k-mer*-based method PopPUNK and the gene-by-gene method with a varying number of loci. PopPUNK is intended for outbreak investigations and rapid identification of clonal strains within bacterial populations, but in our study cgMLST678 split several PPclades, reducing the number of human cases traceable to chicken. PopPUNK, as used here, is more suitable for identifying larger lineages [17]. Moreover, PopPUNK does not remove recombination, either in the core or accessory genome, and recombination erodes the clonal signal leading to introgression of lineages with shared ecology [33]. Contrary to this, the gene-by-gene method efficiently buffers the recombination and is considered a more robust way to identify clonality and sub-populations [34], especially when adapting a proven nomenclature and interpretation rules (as for the cgMLST678 INNUENDO (L1:L2:L3)). L1-cgMLST678 showed a remarkable ability to identify clonal isolates, as only a minority of the clusters were broken up by inclusion of more loci (cgMLST95, cgMLST99 and wgMLST). Thus, the L1-cgMLST678 nomenclature proved to be a robust method to identify disseminated outbreaks in this study, and was shown to be even more discriminatory, albeit also more labor-intensive, than the *k-mer*-based PopPUNK.

To conclude, the use of WGS genotyping and one single metadata value of the collection date allowed us to make deductions on the origin of human cases and trace ten human cases to fresh chicken meat. Although additional data on consumer habits would be needed to confirm this suspicion, we found that the majority of human cases occurring during the summer peak did not share genotypes with a chicken flock slaughtered prior to the occurrence of illness, and fresh chicken meat was therefore an unlikely source for the remaining (81.6%) cases. To more precisely evaluate sources and possible vehicles in Finland, larger, representative sentinel studies simultaneously performing case-control studies, sampling of isolates and WGS analysis from different reservoirs and human cases are needed. Such studies are lacking in the Nordic countries but should be established to elucidate the incompletely understood Nordic *Campylobacter* epidemiology.

## 4. Materials and Methods

### 4.1. Selection of Isolates

Isolates of *C. jejuni* acquired retrospectively from routine stool samples of campylobacteriosis patients from the western region Satakunta (inhabitants 216,752 in 2019 [35]) between July 15 and 1 September 2014, lacking a recent travel history were included in the study (*n* = 50), which represented all reported possible domestic cases (domestic and unknown travel history) during this time period [36]. This region was chosen since many of the Finnish broiler farms are located in Satakunta and it is a middle-sized region consisting of both urban and rural settings [37]. 

*C. jejuni* isolates from the Finnish monitoring program (FMP) for *Campylobacter* [6] collected from chicken flocks slaughtered up to 23 days prior to the reported human campylobacteriosis cases were included as a possible reservoir or source. This timespan was chosen to account for slaughter, time-at-market and incubation period, according to the criteria of Kärenlampi et al. (2003) and used by Kovanen et al. (2016) [12,38]. In addition, we included chicken strains collected between 1–24 September 2014, to catch possible indirect transmission from the chicken to humans. Furthermore, two chicken isolates between September 24 and October 1 were included as an outgroup. We therefore had 55 *C. jejuni* isolates from broiler chickens collected between June 23 and 30 September 2014, acquired through the FMP included in this study. In brief, the FMP samples all chicken batches slaughtered in Finland between June and October, and the detection of *C. jejuni* is done by the direct plating of a pooled cecal sample (10 cecas/batch) on mCCDA according to NMKL No. 119 [39]. A single typical colony was selected for further analysis according to the method of The Food Safety Authority (Ruokavirasto) 3512/5 [7]. A total of 85 *C. jejuni* isolates from 1507 slaughter batches (6.0% positive) were obtained between June and October 2014, of which 61 were isolated during the same period as the human patient isolates (June to September) originating from a total of 37 farms, indicating that a positive farm could have several positive batches (from one to four) [40]. To account for the clustering of *C. jejuni* genotypes from chicken farms, only one isolate was included when several flocks reared at the same time on the same farm were positive for *C. jejuni.* This is in line with the observed clustering genotypes between slaughter batches on the same farm; these usually have a similar MLST, PFGE and wgMLST profile in Finland [12,25]. This selection of isolates therefore represents the entire population of *C. jejuni* available through the consumption and handling of Finnish chicken meat during the summer peak.

### 4.2. DNA Extraction, WGS, Assembly and Multilocus Sequence Typing (MLST)

The isolates were grown on nutrient agar supplied with defibrinated horse/bovine blood (CM0003, Oxoid, ThermoFisher, Vantaa, Finland) under microaerophilic conditions for 18–20 h prior to plate-harvest and DNA extraction using the PureLink™ Genomic DNA Mini Kit (K182001, Invitrogen, ThermoFisher, Vantaa, Finland) or QIAGEN DNeasy Blood & Tissue Kit using the Gram-positive protocol (QIAGEN). DNA quality was assessed by 260/280 and 260/230 ratios by NanoDrop™ 2000/2000c Spectrophotometer (Thermofisher, Vantaa, Finland), and quantity by Qubit Fluorometric Quantification (Thermofisher, Vantaa, Finland) using the Invitrogen™ Qubit™ dsDNA BR (Broad Range) Assay (ThermoFisher, Vantaa, Finland). Paired-end sequencing (read length of 100-bp, 150-bp, or 250-bp) using Nextera XT library preparation (*n* = 39 chicken and all human) or Illumina^®^ DNA Prep (*n* = 5 chicken isolates) (Illumina, San Diego, CA, USA) was carried out according to the manufacturer’s instructions and sequencing was conducted on either the HiSeq platform (Illumina, San Diego, CA, USA) by a commercial provider (Institute for Molecular Medicine Finland (FIMM), Helsinki, Finland) or on the MiSeq platform (platform) in-house (*n* = 5 chicken isolates). Sequencing reads were subjected to quality control, de novo assembly, and MLST definition using INNUca pipelines (version 3.1.1 and 4.2.2-02 (*n* = 5 chicken isolates)) [41]. MLST types were derived from the pubMLST database [20,22].

### 4.3. Analysis of the Clonal Relationship between the Human and Chicken C. jejuni Isolates

PopPUNK (version 2.2.0) [17] was used to analyze the population structure and assign isolates to clusters. PopPUNK uses k-mer comparisons to characterize genomic variation in the core and accessory genome, and therefore exploits the information available in the entire genome; isolates are clustered if the intra-cluster genetic distance is less than the inter-cluster genetic distances. The model was run with the default settings (--easy-run, --plot-fit 5, --min-k 13, --full-db) and the model was refitted using the existing database by increasing the number of mixture components to the number of blobs we judged to be in the plot (*n* = 4). In addition, PopPUNK produces a neighbor joining tree from the core-distances, which was visualized together with the associated metadata on the Microreact online server [19] to make use of the temporal visualization tools embedded there.

To further assess the clonal relationship between the isolates within each cluster, genome assemblies of chicken and human isolates and the remainder of the INNUENDO database (in total 5691 *C. jejuni* genomes were compared using the cgMLST schema from INNUENDO with 678 loci (cgMLST678) [16,18] through the use of the chewBBACA suite (version 2.5.5) [21]. Minimum spanning trees (MST) and goeBurst distances were calculated using the goeBURST Full MST algorithm implemented in the desktop version of PHYLOViZ 2.0 [42,43], and used to define cgMLST678 profiles L1:L2:L3 [16]. The nomenclature representing the highest resolution, L1, allows up to four loci variations, and is intended for outbreak investigation, while L2 is a robust threshold for quasi-stable clustering, and L3 generally corresponds to MLST [44]. To offer increased resolution of the clusters forming in the cgMLST678 analysis, wgMLST profiles based on the INNUENDO wgMLST schema for the isolates were defined by the chewBBACA suite, and were subsequently used to define cgMLST profiles at two different levels; present in at least 95% (cgMLST95) and 99% (cgMLST99) of the strains [16]. Identical L1:L2:L3 profiles were used as an initial cut-off to identify potential clusters, which were reinvestigated using cgMLST99 and cgMLST95 with a cut-off of six allowed variable loci (0.59% variation allowed). To classify as temporal association between clustering isolates, the chicken isolates had to have been collected between 2–23 days prior to the human isolates, accommodating time for slaughter, time-on-market and incubation time.

## Figures and Tables

**Table 1:** Number of isolates from each source and their respective sequence types (STs) according to the 7-loci multilocus sequence typing (MLST) schema [22].

ST	Human	Chicken	Human Cases Preceded by Chicken	Temporal Human Clusters	Total
45	19	13	18	All	32
677	5	4	5	1	9
122	0	6	NA	NA	6
267	5	2	3	2	7
11	1	6	0	0	7
794	4	0	0	1	4
230	1	2	1	NA	3
538	1	2	0	0	3
21	1	3	1	0	4
19	1	1	1	NA	2
227	0	2	NA	NA	2
383	0	2	NA	NA	2
2219	0	2	0	0	2
Other *	11	10	0	0	21

* “Other” refers to STs represented with only one isolate.

**Table 2:** Overview of isolates clustering together with four or fewer allele differences according to the cgMLST678 schema, i.e., having similar L1-cgMLST678 types.

ST	Isolates *n* (Human/Chicken)	Allele Difference ^a^	Collection Dates Human	Collection Dates Chicken	L1:L2:L3
**Chicken Before Human Clusters**					
19	1/1	0	August 31	July 17	16:13:8
21	1/2	0	August 26 ^c^	August 14 ^c^ and August 20 ^c^	31:25:3
45	2/1	0	July 21 ^c^ and July 31	July 17 ^c^	2:6:1
230	1/1	0	August 4	July 23	8:8:1
267	2/1	0	August 11 and August 12	July 28	10:4:1
538 ^b^	1/1	0	July 18	June 25	53:38:1
677	1/1	0	July 19 ^c^	July 4 ^c^	11:3:2
677	1/1	0	August 13	July 31	62:3:2
**Human Clusters**					
45 ^b^	2	3	July 21 and July 23	NA	7:2:1
45	2	0	July 22 and July 29	NA	13:6:1
**Humans Before/Simultaneous as Chicken**					
45	1/1	2	July 18	July 28	5:2:1
45 ^b^	1/1	3	September 1	September 19	3:1:1
267	2/1	0	August 11 ^c^ and August 12	August 12 ^c^	10:4:1
538	1/1	0	July 18	August 11	53:38:1
**Chicken Clusters**					
2219	2	0	NA	July 18 and July 21	44:5:1
11 ^b^	3	2	NA	July 3, July 21 and July 23	1:5:1
11	3	0	NA	July 28 and August 11	44:31:1
45	2	0	NA	August 18 and August 22	37:2:1
383	2	0	NA	August 20 and August 22	49:34:1
122 and 755	6	0	NA	September 10 and September 24	4:9:4

^a^ According to the cgMLST678 profile; ^b^ clusters breaking in the cgMLST99 or cgMLST95 analysis; ^c^ wgMLST profile also similar.

## Supplementary Materials

The following are available online at https://www.mdpi.com/2076-0817/9/11/868/s1, Table S1: Assemblies, source and date of the isolates, and associated genotyping results.

## References

[1] Scarborough P., Appleby P.N., Mizdrak A., Briggs A.D.M., Travis R.C., Bradbury K.E., Key T.J. (2014). Dietary greenhouse gas emissions of meat-eaters, fish-eaters, vegetarians and vegans in the UK. Clim. Chang..

[2] Suomen Siipikarjaliitto Ry Siipikarjatuotanto *Suomessa 1995–2019*. http://www.siipi.net/index.php/siipikarjaliitto/tilastoa.

[3] EFSA Panel on Biological Hazards (BIOHAZ) (2010). Scientific Opinion on Quantification of the risk posed by broiler meat to human campylobacteriosis in the EU. EFSA J..

[4] Romero-Barrios P., Hempen M., Messens W., Stella P., Hugas M. (2013). Quantitative microbiological risk assessment (QMRA) of food-borne zoonoses at the European level. Food Control.

[5] WHO (World Health Organization), OIE (World Organisation for Animal Health) (2013). The global view of campylobacteriosis: Report of an expert consultation. WHO Report.

[6] Maa-Ja Metsätalousministeriö (2007). Maa-Ja Metsätalousministeriön Asetusbroilereiden Kampylobakteerivalvonnasta.

[7] Gonzalez M., Mikkelä A., Tuominen P., Ranta J., Hakkinen M., Hänninen M.-L., Llarena A.-K. (2016). Risk Assessment of Campylobacter spp. in Finland. Evira Research Report.

[8] Jaakola S.L., Rimhanen-Finne R., Salmenlinna S., Savolainen-Kopra C., Liitsola K., Jalava J., Toropainen M., Nohynek H., Virtanen M., Löflund J.-E. (2018). Infectious Diseases in Finland 2017.

[9] Rosef O., Paulauskas A., Grude N., Haslekås C., Jenkins A. (2009). Comparison of Norwegian poultry, waterborne and clinical isolates of Campylobacter jejuni by ribotyping. J. Bacteriol..

[10] Kapperud G., Espeland G., Wahl E., Walde A., Herikstad H., Gustavsen S., Digranes A. (2003). Factors Associated with Increased and Decreased Risk of Campylobacter Infection: A Prospective Case-Control Study in Norway. Am. J. Epidemiol..

[11] Schönberg-Norio D., Takkinen J., Hänninen M.-L., Katila M.-L., Kaukoranta S.-S., Mattila L., Rautelin H. (2004). Swimming and Campylobacter infections. Emerg. Infect. Dis..

[12] Kovanen S., Kivistö R., Llarena A.K., Zhang J., Kärkkäinen U.M., Tuuminen T., Uksila J., Hakkinen M., Rossi M., Hänninen M.L. (2016). Tracing isolates from domestic human Campylobacter jejuni infections to chicken slaughter batches and swimming water using whole-genome multilocus sequence typing. Int. J. Food Microbiol..

[13] Castrodale L.J., Provo G.M., Xavier C.M., McLaughlin J.B. (2016). Calling all Campy—how routine investigation and molecular characterization impacts the understanding of campylobacteriosis epidemiology—Alaska, United States, 2004–2013. Epidemiol. Infect..

[14] Strachan N.F., Forbes K.J., Sheppard S. (2014). Extensive Spatial and Temporal Clustering of Campylobacter Infections Evident in High-Resolution Genotypes. Campylobacter Ecology and Evolution.

[15] Joensen K.G., Kuhn K.G., Muller L., Bjorkman J.T., Torpdahl M., Engberg J., Holt H.M., Nielsen H.L., Petersen A.M., Ethelberg S. (2018). Whole-genome sequencing of Campylobacter jejuni isolated from Danish routine human stool samples reveals surprising degree of clustering. Clin. Microbiol. Infect..

[16] Llarena A.-K., Ribeiro-Gonçalves B.F., Nuno Silva D., Halkilahti J., Machado M.P., Da Silva M.S., Jaakkonen A., Isidro J., Hämäläinen C., Joenperä J. (2018). INNUENDO: A cross-sectoral platform for the integration of genomics in the surveillance of food-borne pathogens. EFSA Supporting Publ..

[17] Lees J.A., Harris S.R., Tonkin-Hill G., Gladstone R.A., Lo S.W., Weiser J.N., Corander J., Bentley S.D., Croucher N.J. (2019). Fast and flexible bacterial genomic epidemiology with PopPUNK. Genome Res..

[18] Rossi M., Da Silva M.S., Ribeiro-Gonçalves B.F., Silva D.N., Machado M.P., Oleastro M., Borges V., Isidro J., Viera L., Barker D. (2018). NNUENDO whole genome and core genome MLST schemas and datasets for Campylobacter jejuni (Version 1.0) [Data set]. Zenodo.

[19] Argimón S., Abudahab K., Goater R.J.E., Fedosejev A., Bhai J., Glasner C., Feil E.J., Holden M.T.G., Yeats C.A., Grundmann H. (2016). Microreact: Visualizing and sharing data for genomic epidemiology and phylogeography. Microb. Genome.

[20] Dingle K.E., Colles F.M., Wareing D.R.A., Ure R., Fox A.J., Bolton F.E., Bootsma H.J., Willems R.J.L., Urwin R., Maiden M.C.J. (2001). Multilocus Sequence Typing System forCampylobacter jejuni. J. Clin. Microbiol..

[21] Silva M., Machado M.P., Silva D.N., Rossi M., Moran-Gilad J., Santos S., Ramirez M., Carriço J.A. (2018). chewBBACA: A complete suite for gene-by-gene schema creation and strain identification. Microb. Genome.

[22] Jolley K.A., Bray J.E., Maiden M.C. (2018). Open-access bacterial population genomics: BIGSdb software, the PubMLST.org website and their applications. Wellcome Open Res..

[23] Neimann J., Engberg J., Molbak K., Wegener H.C. (2003). A case-control study of risk factors for sporadic campylobacter infections in Denmark. Epidemiol. Infect..

[24] MacDonald E., White R., Mexia R., Bruun T., Kapperud G., Lange H., Nygard K., Vold L. (2015). Risk Factors for Sporadic Domestically Acquired Campylobacter Infections in Norway 2010-2011: A National Prospective Case-Control Study. PLoS ONE.

[25] Llarena A.-K., Huneau A., Hakkinen M., Hänninen M.-L. (2015). Predominant Campylobacter jejuni sequence types persist in Finnish chicken production. PLoS ONE.

[26] De Haan C.P., Kivistö R.I., Hakkinen M., Corander J., Hänninen M.-L.J.B.M. (2010). Multilocus sequence types of Finnish bovine Campylobacter jejuni isolates and their attribution to human infections. BMC Microbiol..

[27] Smith S., Messam L.L.M., Meade J., Gibbons J., McGill K., Bolton D., Whyte P. (2016). The impact of biosecurity and partial depopulation on Campylobacter prevalence in Irish broiler flocks with differing levels of hygiene and economic performance. Infect. Ecol. Epidemiol..

[28] Llarena A.-K., Zhang J., Vehkala M., Välimäki N., Hakkinen M., Hänninen M.-L., Roasto M., Mäesaar M., Taboada E., Barker D. (2016). Monomorphic genotypes within a generalist lineage of Campylobacter jejuni show signs of global dispersion. Microb. Genome.

[29] Lihatiedotusyhdistys, Ry Lihatuotanto Suomessa.

[30] Elvers K.T., Morris V.K., Newell D.G., Allen V.M. (2011). Molecular Tracking, through Processing, of Campylobacter Strains Colonizing Broiler Flocks. Appl. Environ. Microbiol..

[31] Jonsson M.E., Chriél M., Norström M., Hofshagen M. (2012). Effect of climate and farm environment on Campylobacter spp. colonisation in Norwegian broiler flocks. Prev. Vet. Med..

[32] Tam C.C., Rodrigues L.C., Viviani L., Dodds J.P., Evans M.R., Hunter P.R., Gray J.J., Letley L.H., Rait G., Tompkins D.S. (2012). Longitudinal study of infectious intestinal disease in the UK (IID2 study): Incidence in the community and presenting to general practice. Gut.

[33] Sheppard S.K., Maiden M.C.J. (2015). The Evolution of Campylobacter jejuni and Campylobacter coli. Cold Spring Harb. Perspect. Biol..

[34] Maiden M.C.J., van Rensburg M.J.J., Bray J.E., Earle S.G., Ford S.A., Jolley K.A., McCarthy N.D. (2013). MLST revisited: The gene-by-gene approach to bacterial genomics. Nat. Rev. Microbiol..

[35] Satakuntoliitto. http://www.satakunta.fi/.

[36] THL Tartuntatautirekisterin Tilastotietokanta. https://www.thl.fi/ttr/gen/rpt/tilastot.html.

[37] Suomen Broileryhdistys, Ry Missä Broilerituotantoa on?. http://suomibroileri.fi/fi/missa.

[38] Kärenlampi R., Rautelin H., Hakkinen M., Hänninen M.L. (2003). Temporal and geographical distribution and overlap of Penner heat-stable serotypes and pulsed-field gel electrophoresis genotypes of Campylobacter jejuni isolates collected from humans and chickens in Finland during a seasonal peak. J. Clin. Microbiol..

[39] Institute N.F. (2007). Thermotolerant Campylobacter. Detection, Semi-Quantitative and Quantitative Determination in Foods and Drinking Water.

[40] European Food Safety Authority and European Centre for Disease Prevention and Control (ECDC) (2015). The European Union summary report on trends and sources of zoonoses, zoonotic agents and food-borne outbreaks in 2014. EFSA J..

[41] Machado M.P.H., Halkilahti J., Jaakkonen A., Silva D.N., Mendes I., Nalbantoglu Y., Borges V., Ramirez M., Rossi M., Carriço J.A. INNUca GitHub. https://github.com/B-UMMI/INNUca.

[42] Francisco A.P., Bugalho M., Ramirez M., Carriço J.A. (2009). Global optimal eBURST analysis of multilocus typing data using a graphic matroid approach. BMC Bioinform..

[43] Nascimento M., Sousa A., Ramirez M., Francisco A.P., Carriço J.A., Vaz C. (2016). PHYLOViZ 2.0: Providing scalable data integration and visualization for multiple phylogenetic inference methods. Bioinformatics.

[44] Carriço J.A., Silva-Costa C., Melo-Cristino J., Pinto F.R., de Lencastre H., Almeida J.S., Ramirez M. (2006). Illustration of a Common Framework for Relating Multiple Typing Methods by Application to Macrolide-Resistant Streptococcus pyogenes. J. Clin. Microbiol..

